# Compositional Data Analysis of Microbiome and Any-Omics Datasets: A Validation of the Additive Logratio Transformation

**DOI:** 10.3389/fmicb.2021.727398

**Published:** 2021-10-11

**Authors:** Michael Greenacre, Marina Martínez-Álvaro, Agustín Blasco

**Affiliations:** ^1^Department of Economics and Business, Universitat Pompeu Fabra, Barcelona, Spain; ^2^Department of Agriculture, Horticulture and Engineering Sciences, Scotland's Rural College, Edinburgh, United Kingdom; ^3^Institute for Animal Science and Technology, Universitat Politècnica de València, València, Spain

**Keywords:** compositional data, dimension reduction, logratio transformation, logratio geometry, logratio variance, Procrustes correlation, variable selection

## Abstract

Microbiome and omics datasets are, by their intrinsic biological nature, of high dimensionality, characterized by counts of large numbers of components (microbial genes, operational taxonomic units, RNA transcripts, etc.). These data are generally regarded as compositional since the total number of counts identified within a sample is irrelevant. The central concept in compositional data analysis is the logratio transformation, the simplest being the additive logratios with respect to a fixed reference component. A full set of additive logratios is not isometric, that is they do not reproduce the geometry of all pairwise logratios exactly, but their lack of isometry can be measured by the Procrustes correlation. The reference component can be chosen to maximize the Procrustes correlation between the additive logratio geometry and the exact logratio geometry, and for high-dimensional data there are many potential references. As a secondary criterion, minimizing the variance of the reference component's log-transformed relative abundance values makes the subsequent interpretation of the logratios even easier. On each of three high-dimensional omics datasets the additive logratio transformation was performed, using references that were identified according to the abovementioned criteria. For each dataset the compositional data structure was successfully reproduced, that is the additive logratios were very close to being isometric. The Procrustes correlations achieved for these datasets were 0.9991, 0.9974, and 0.9902, respectively. We thus demonstrate, for high-dimensional compositional data, that additive logratios can provide a valid choice as transformed variables, which (a) are subcompositionally coherent, (b) explain 100% of the total logratio variance and (c) come measurably very close to being isometric. The interpretation of additive logratios is much simpler than the complex isometric alternatives and, when the variance of the log-transformed reference is very low, it is even simpler since each additive logratio can be identified with a corresponding compositional component.

## Introduction

The *Frontiers in Microbiology* article by Gloor et al. (2017) is emphatically titled: “Microbiome datasets are compositional: and this is not optional.” We agree. For example, the number of so-called reads obtained by high throughput sequencing varies from sample to sample and is of no relevance to the investigation, much the same as the size of a rock is irrelevant to the study of its geochemical composition. It is the relative values of the read counts that are the data of interest, thus making the data strictly compositional (Fernandes et al., 2014). The same is true for other assay methods such as liquid chromatography–mass spectrometry where identification of metabolites is achieved by intensity values or integrated areas under peaks.

It is convenient to eliminate the effect of the sample totals by normalizing, or *closing*, the data, so that sample values sum to 1—these vectors of non-negative sample values with constant sums are called *compositions*. Once this initial step is made, the question remains how to analyze, relate and interpret the *components* of the compositions, be they microbial genes, operational taxonomic units, transcripts or metabolites. The essential decision is whether it makes sense to use these relative abundances in the statistical analysis or some transformed version of them. This is the first and most fundamental step in the pipeline for analyzing compositional data.

It has long been appreciated, since the pioneering work of John Aitchison (Aitchison, 1982, 1986, 1997), that a valid, *(subcompositionally) coherent* way to tackle compositional data is by considering pairwise ratios of the components and by analyzing these ratios after logarithmic transformation. Notice that these ratios are invariant with respect to the normalization (closure) of the data. Coherence means that, if the set of components is extended or reduced, the ratios of the common components remain constant in spite of the changing values of their relative abundances. In fact, the set of components under consideration, imposed by the measuring instrument, research objective and practical considerations, is almost always a subset of a potentially much larger set.

The basic concept and data transformation in compositional data analysis is thus the logratio, the logarithm of pairwise ratios, with the log-transformation serving several purposes:

1. Taking the data into real space,
2. Turning interval differences on the log-scale into percentage differences when back-transformed to the original ratio scale,
3. Symmetrizing the positively skew distributions of the ratios, and
4. Making more meaningful the application of interval-based statistical summaries and analyses, such as variance, Euclidean distance, regression and dimension reduction.

The challenge is to choose a data transformation that replaces the compositional dataset with a set of logratios that are substantively meaningful to the practitioner as well as having a clear interpretation. Once the transformation to logratios is performed, analysis, visualization and inference carries on as before, but always taking into account the interpretation in terms of ratios.

In Aitchison's earliest work he proposed the additive logratio transformation (ALR), where one component is chosen as the denominator, or *reference*, with all the other components as numerators. Thus, if there are *J* components, with values *X*_1_, *X*_2_, …, *X*_*J*_, there are *J* − 1 logratios in the ALR set with respect to the selected reference component, denoted by *ref* , of the form:


(1) $$\begin{array}{l} \text{ALR}\left(j|\text{ref}\right)=\log\left(X_{j}/X_{\text{ref}}\right),\;j=1,\ldots ,J,\;j\neq \text{ref} \end{array}$$

Since then a variety of logratio transformations have been proposed: for example, centered logratios (used by Sisk-Hackworth and Kelley, 2020), isometric logratios and pivot logratios (for example, Pawlowsky-Glahn and Buccianti, 2011; Filzmoser et al., 2018). All of these involve ratios of geometric means of components and, as a result, have complicated interpretations (Greenacre et al., 2020; Hron et al., 2021), lacking the simplicity of the pairwise logratio between two components. Isometric and pivot logratios are particularly problematic when the numbers of components in the geometric means are high. They do have the property of isometry, however, which means that they engender exactly the same multivariate geometric structure of the sample points as that of all the pairwise logratios, called the *logratio geometry* (sometimes referred to as the “Aitchison geometry”). The proponents of these complex transformations take isometry as a type of “gold standard” for the analysis of compositional data, and the strict adherence to this mathematical ideal has been to the detriment of using simpler transformations such as the ALRs, or a subset of pairwise logratios. In a series of papers by Greenacre (2019), Graeve and Greenacre (2020), and Wood and Greenacre (2021) it is shown in a variety of contexts that a set of simple pairwise logratios can satisfactorily approximate the logratio geometry, coming sufficiently close to being isometric for all practical purposes. A tiny loss of isometry is thus traded off in favor of the benefit of the simpler and clearer interpretation of the logratio variables. In these above-mentioned studies any set of pairwise logratios can be selected, whereas ALRs are restricted to pairwise logratios with respect to a fixed reference component.

Apart from the fact that ALRs are not strictly isometric, various other criticisms have been leveled at the ALR transformation, such as its sacrificing a component to serve as the reference and the doubt about which component to choose as reference. We hope to show that none of the above are disadvantages, but rather that, especially in the case of high-dimensional compositional data, the ALRs are the logratio transformations of choice and that their involving a fixed reference is actually a benefit. In this way we return to the origins of compositional data analysis and re-establish the additive logratio in all fields of omics research, thereby vindicating Aitchison's original claim as enounced in the following quotation from his keynote address (Aitchison, 2008) at the biennial Compositional Data Analysis workshop in 2008 (section 5.1):

“The ALR transformation methodology has, in my view, withstood all attacks on its validity as a statistical modeling tool. Indeed, it is an approach to practical compositional data analysis which I recommend particularly for non-mathematicians. The advantage of its logratios involving only two components, in contrast to CLR and ILR (isometric transformations .), which use logratios involving more than two and often many components, makes for simple interpretation and far outweighs any criticism, more imagined than real, that the transformation is not isometric.”

Aitchison's phrasing above that the criticism of the ALR transformation not being isometric is “more imagined than real,” is particularly pertinent to what we will show here. We will demonstrate quantitatively that a set of ALRs can be so close to being isometric that, for all practical purposes, they are isometric. We will also show that there are clearly defined criteria for choosing a reference and it is advantageous that there are very many potential choices in high-dimensional data when the number of components is large.

Three high-dimensional omics datasets will be used to show that the ALR transformation can validly provide a set of simple variables to represent the whole compositional dataset, the essential step being the choice of the reference component. The next section gives some background theoretical material, and details the computational steps involved in determining and validating the chosen ALRs. Then there is a section with results for each of the datasets, and two closing sections with discussion and potential implications for practitioners.

## Methods

Logratio-based compositional data analysis, often called CoDA (Pawlowsky-Glahn and Buccianti, 2011), has mainly developed in fields where the number of components *J* is less, often much less, than the number of samples *I*, i.e., *J* < *I*, with geochemistry being the area of most applications. A short, yet comprehensive, review of CoDA is given by Greenacre (2021), with recent books aimed at practitioners by Filzmoser et al. (2018) and Greenacre (2018). The relevant theoretical results for our purpose are summarized in this section, as well as how they apply to ALRs.

### Total Logratio Variance

The total logratio variance is a basic statistic that quantifies how dispersed the samples are in the multivariate logratio space. A compositional data vector with *J* components, *X*_1_, *X*_2_, …, *X*_*J*_, can be expanded into $\frac{1}{2}J\left(J-1\right)$ pairwise ratios, and then log-transformed. Thus, an *I* × *J* compositional data matrix can be expanded, notionally at least, to an $I\times \frac{1}{2}J\left(J-1\right)$ matrix of logratios. In the most general case, there are positive weights *c*_1_, *c*_2_, …, *c*_*J*_ associated with the components (Lewi, 1976, 1986, 2005; Greenacre and Lewi, 2009), where *c*_1_+*c*_2_+⋯+*c*_*J*_ = 1, in which case it can be shown that the (*j, k*)-th logratio log(*X*_*j*_/*X*_*k*_) has weight equal to the product *c*_*j*_*c*_*k*_ (Greenacre, 2018, 2021). The total logratio variance is then defined as the weighted sum of pairwise logratio variances:


(2) $$\begin{array}{l} \text{TotVar}=\underset{j<k}{\sum \sum }c_{j}c_{k}{\text{Var}}_{jk} \end{array}$$

where Var_*jk*_ is the variance of the (*j, k*)-th logratio (Greenacre, 2018, 2021). The weights have a normalizing function to balance out the contributions of the different components, since rarer components often engender excessively large logratio variances (Fernandes et al., 2014; Greenacre, 2018; Quinn et al., 2019), or they might be used to downweight components with high measurement error. However, in many applications, including the ones in this article, this aspect is ignored and the components are equally weighted by *c*_*j*_ = 1/*J*, *j* = 1, …, *J*. Consequently, Equation (2) simplifies as the sum of the $\frac{1}{2}J\left(J-1\right)$ variances of the unique pairwise logratios multiplied by 1/*J*^2^.

For a dataset with thousands of components this would be a laborious calculation, but fortunately there is a shortcut thanks to the centered logratio (CLR) transformation:


(3) $$\begin{array}{l} \text{CLR}\left(j\right)=\log\left(\frac{X_{j}}{g\left(X\right)}\right),\;j=1,\ldots ,J \end{array}$$

where *g*(*X*) is the weighted geometric mean $X_{1}^{c_{1}}X_{2}^{c_{2}}\cdots X_{J}^{c_{J}}$ (Greenacre, 2018), that is


(4) $$\begin{array}{l} \begin{array}{c} \text{CLR}\left(j\right)=\log\left(X_{j}\right)-\overset{J}{\underset{k=1}{\sum }}c_{k}\log\left(X_{k}\right)\;\left(\text{weighted case}\right)\\ \;=\log\left(X_{j}\right)-\frac{1}{J}\overset{J}{\underset{k=1}{\sum }}\log\left(X_{k}\right)\;\left(\text{unweighted case}\right) \end{array} \end{array}$$

The total variance in (2) is then equivalently computed using the variances of the CLRs, Var_*j*_, weighted, respectively by *c*_*j*_, *j* = 1, …, *J*, or by constant 1/*J* when equally weighted:


(5) $$\begin{array}{l} \begin{array}{c} \text{TotVar}=\overset{J}{\underset{j=1}{\sum }}c_{j}{\text{Var}}_{j}\;\left(\text{weighted case}\right)\\ \;=\frac{1}{J}\overset{J}{\underset{j=1}{\sum }}{\text{Var}}_{j}\;\left(\text{unweighted case}\right) \end{array} \end{array}$$

Notice that in the weighted or unweighted cases the CLRs have to be computed according to one of the respective definitions in Equation (4). Notice too that Equations (2), (5), with either differential or equal weights, are weighted averages of the part variances, ensuring that total logratio variances can be compared between data sets of different sizes.

The computation is completely symmetric with respect to rows and columns, so when *J* > *I*, as will generally be the case for omics data, the computation can be further simplified. The data matrix is first transposed and relative abundances are expressed with respect to component totals, then repeating the above computation as if the samples were the components gives identical results (Greenacre, 2018).

### Logratio Geometry

A compositional dataset has a certain exact geometry defined by the logratio distances between every pair of samples. These are Euclidean distances that can be defined in two equivalent ways: either on the $I\times \frac{1}{2}J\left(J-1\right)$ matrix of all pairwise logratios, again a very wide matrix due to the large number of pairs of components, or more efficiently on the *I* × *J* matrix of CLRs (4). As before, there are weighted and unweighted versions—for the exact definitions see Greenacre (2018, 2021). If *J* < *I* (i.e., the dataset is “narrow”) the sample points are exactly in a (*J* − 1)-dimensional Euclidean space, otherwise if *J* > *I* (i.e., the dataset is “wide”) they are exactly contained in a (*I* − 1)-dimensional Euclidean space—hence, the dimensionality is *K* = min{*I* − 1, *J* − 1}.

In both weighted and unweighted cases the total logratio variance can be decomposed along principal axes to give a low-dimensional reduced view of the samples, called *logratio analysis* (LRA) (Greenacre and Lewi, 2009; Greenacre, 2010). LRA is the principal component analysis (PCA) of all the pairwise logratios, which is equivalent to the PCA of all the CLRs, in weighted (Greenacre and Lewi, 2009) or unweighted (Aitchison and Greenacre, 2002) forms.

Notice that for a compositional data set of dimensionality *J* − 1, say (for the case *J* ≤ *I*), then any set of *J* − 1 linearly independent logratios, including any set of *J* − 1 ALRs, explains the total logratio variance in (2) or (5) completely. This set clearly does not contain the total variance, but explains it totally in a regression sense (Greenacre, 2019). If *J* > *I*, as in many high-dimensional datasets, only *I* − 1 linearly independent logratios are required to explain fully the total logratio variance.

### Procrustes Analysis

For any particular set of logratio transformations, the samples in the transformed space can be “fitted" to the exact logratio geometry, using *Procrustes analysis* (Gower and Dijksterhuis, 2004; Lisboa et al., 2014), to see how close they come to the exact geometry. Suppose the coordinates of the samples in their exact logratio geometry are in the matrix *X* (*I* × *K*), where *K* is the dimensionality of the space, as explained above. The coordinates are established using LRA and the inter-sample distances in this geometry are exactly the logratio distances. Similarly, suppose the coordinates of the samples in a particular ALR geometry are in the matrix *Y* (*I* × *K*), the same dimensionality as the exact one—for example, if *J* > *I* (as in the present case) then the dimensionality of the logratio space is *K* = *I* − 1 (one less than the number of samples), and that of the *J* − 1 ALRs, also involving *I* samples, is also *I* − 1. The sample coordinates in the ALR geometry are established using PCA and the inter-sample distances in this ALR geometry will not be the same as the exact logratio distances, partly due to differences in scale and rotation between the two matrices, which are irrelevant to summarizing their distance structure. So Procrustes analysis aims to match the configurations by least squares as closely as possible by three simple operations: centering, scaling and rotation.

The first two operations are trivial: the columns of *X* and *Y* are already centered by the LRA and PCA, respectively, and scaling is achieved by dividing each matrix by the square roots of their respective sum-of-squares. Suppose X^*^ and Y^*^ are the matrices standardized in this way, then compute the singular value decomposition of their cross-product (^X^*^)*T*^Y^*^ = *UDV*^*T*^. The fitting of Y^*^ to X^*^ by least-squares fitting is achieved by applying the rotation matrix *Q* = *VU*^*T*^ to Y^*^: Y^*^Q. Equivalently, X^*^ could be fitted to Y^*^ by applying the inverse rotation *Q*^*T*^ : X^*^Q^*T*^.

The final step is to compute the Procrustes correlation, which measures how close the two configurations are to being exactly matched. The sum-of-squares *E* of the differences between X^*^ and Y^*^Q lies between 0 and 1, where 0 implies perfect matching and 1 implies total absence of matching. The quantity *E* can be considered a residual sum-of-squares if one thinks of Y^*^ being fitted to X^*^, and since *E* has a maximum of 1, then 1 − *E* is analogous to a coefficient of determination (*R*^2^) in a least-squares regression. The Procrustes correlation is thus defined as $R=\sqrt{1-E}$, so that a value near 1 would mean that the ALR geometry is very close to the exact logratio geometry, that is it is almost *isometric*. The Procrustes correlation *R* can be equivalently computed as the regular Pearson correlation between the elements of the matrices X^*^ and Y^*^Q strung out as *IK* × 1 vectors.

In short, the goal is to measure the deviation of the ALR-transformed data from the ideal of isometry. This way of measuring the proximity by the Procrustes correlation between two configurations in multidimensional space has already been used to select a subset of pairwise logratios that engenders a Euclidean geometry close to the exact one (Greenacre, 2019; Graeve and Greenacre, 2020; Wood and Greenacre, 2021). This idea was inspired by the selection of variables in PCA by Krzanowski (1987), and the same idea will be used here to select a reference in order to define a set of ALRs.

### Criteria for Selecting the Reference Component of the Additive Logratios

The ALR transformation converts the original *I* × *J* compositional data matrix to an *I* × (*J* − 1) matrix of ALRs, with respect to a particular reference component. There are *J* potential reference components to choose from, which in the usual geo- and biochemical applications can be a relatively low number. However, in the case of most omics data, *J* is very large and usually very much larger than *I*, the number of samples. This gives a large set of possibilities for choosing a set of ALRs that comes as close as possible to reproducing the exact logratio geometry by achieving a very high Procrustes correlation.

The matching of the geometries is the most important criterion for choosing the reference, but there are other properties that would be beneficial. For example, it would be very convenient if the reference's relative abundances across the samples were as constant as possible. From Equation (1), ALR(*j*|*ref*) = log(*X*_*j*_) − log(*X*_*ref*_), hence we should look for low variance in log(*X*_*ref*_). Since dividing each component by an almost constant reference value just shifts all the logratios by an almost constant amount, the logratio can then be interpreted in practice as its numerator on a logarithmic scale. An additional benefit of choosing a low variance component is that it is unlikely to be correlated with any continuous or categorical covariate whose relationship with the compositions is being investigated—the actual relationship with such covariates can be checked where applicable.

A further criterion would be to avoid choosing a reference with low abundances across samples or with many zeros (that is, low occupancy), where low occupancy is related to low overall abundance (Gaston et al., 2000). Zeros would need to be replaced before making the logratio transformation, using one of the many zero replacement methods, and using such a component as the denominator would affect the interpretation of all the ALRs.

### Validating the ALR Transformation on Three Datasets

Three datasets with high numbers of components are considered here:

- A wide functional microbe dataset of secum samples of *I* = 89 rabbits, in a study of *J* = 3, 937 microbial genes, which we will refer to as the Rabbits data (Martínez-Álvaro et al., 2021b);
- A wide dataset of *I* = 28 mice in a study of *J* = 3147 mRNA transcripts from bone marrow dendritic cells by Jovanovic et al. (2015), re-analyzed by Quinn et al. (2019), which we will refer to as the Mice data;
- A narrower dataset, consisting of spectral data produced by nuclear magnetic resonance (NMR) as part of a study about methane emissions from cattle (Bica et al., 2020), reanalyzed by Štefelová et al. (2021); specifically from *I* = 211 rumen samples measuring *J* = 127 NMR intensities in the form of integrals, which we will refer to as the Cows data. In addition, methane yield (CH4 in gms/kg of dry matter intake) was measured for each individual animal using respiration chambers and diet type was recorded (either concentrate, mixed, or forage-based diet).

For each dataset the following statistics are computed:

(a) The total logratio variance, which is a statistic that summarizes how dispersed the sample points are in multidimensional space (equal weighting of components will be used throughout). For the first two wide examples, the total variance can be more efficiently computed by transposing the matrix of abundances (or relative abundances) and then computing the total variance on the CLRs of the samples, as if they were the components. The exact logratio geometric structure is then determined, that is the coordinates of all the sample points in the full-dimensional space.
  And then, for each component used as a reference for defining ALRs:
(b) The Procrustes correlation between the exact logratio geometry and the approximate geometry of the set of ALRs using the reference;
(c) The variance of the log-transformed relative abundances of the reference candidate across the samples.

The components with the highest correlations in (b) and, of those, the lowest variances in (c) will be candidates for the choice of reference. In practice, of course, domain knowledge should also play a role in selecting the reference, especially when there are several competing candidates.

Finally, having decided on the reference, the reduced-dimension LRA of the exact sample configuration based on all pairwise logratios is shown alongside the reduced-dimension configuration of the chosen set of ALRs to demonstrate that the configurations are practically identical.

## Results

### The Rabbits Data

This is a 89 × 3, 937 dataset of counts and there are no zeros.

**Figure 1:**
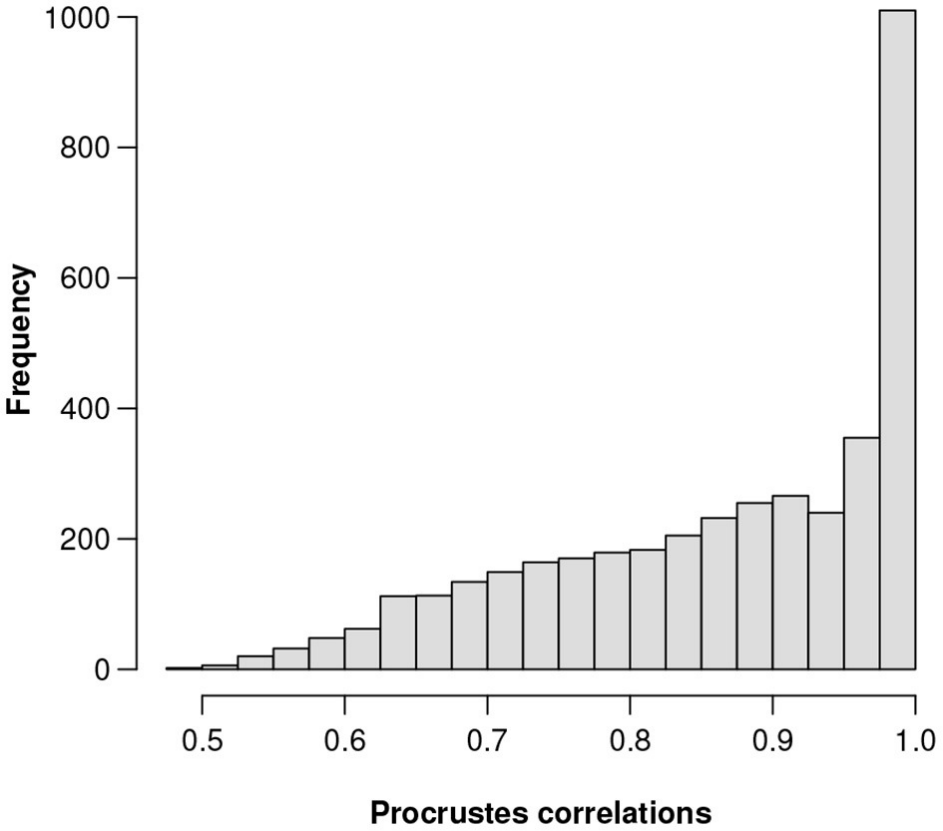
Histogram of the 3,937 Procrustes correlations of the respective sets of candidate ALRs, each set computed using a different reference component.

(a) Total logratio variance = 0.1601, computed on the 3,937 CLRs of the components (microbial genes). Equivalently, a faster way is to transpose the dataset and then treat the samples as components—the same result is obtained on the 89 CLRs of the samples.
(b) The highest Procrustes correlation is equal to 0.9991, corresponding to gene number 856. This gene has the 201st highest relative abundance among the 3,937 genes. Figure 1 shows a histogram of the Procrustes correlations for all 3,937 references.
(c) The lowest variance of the log-transformed relative abundance of the reference components is equal to 0.00117, corresponding to the same gene number 856. Its five-point summary on the log-scale is:

$$\begin{array}{l} \text{ minimum}=-6.97\text{ first quartile}=-6.89\\ \text{ median}=-6.87\text{ third quartile}=-6.84\\ \text{maximum}=-6.76 \end{array}$$

showing a high constancy in the values, with interquartile range of 0.05.

**Figure 2:**
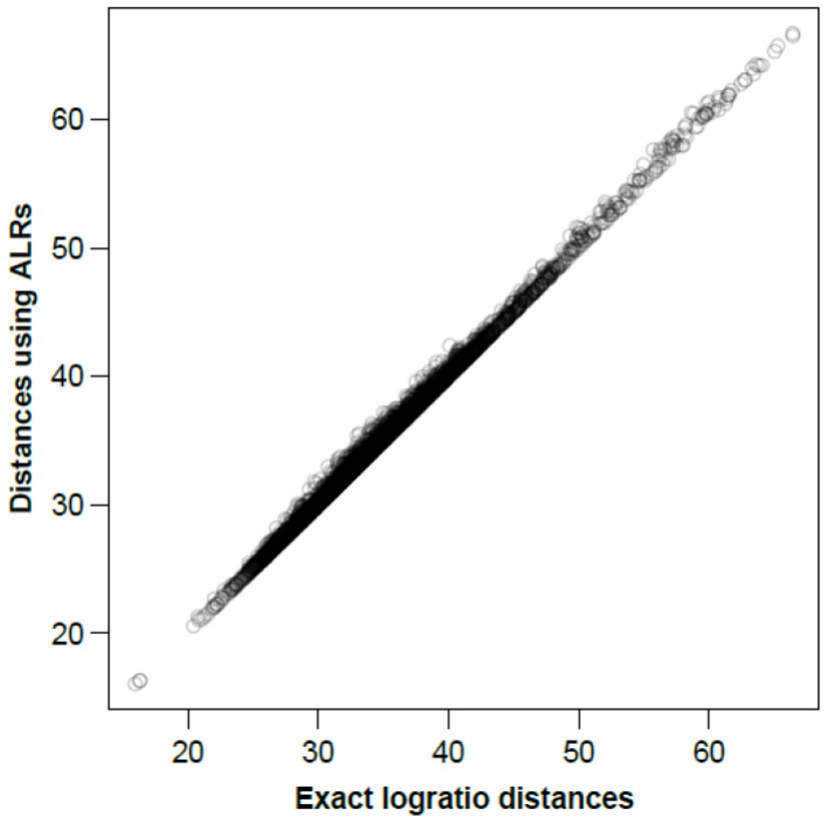
Between-sample distances for the Rabbits dataset based on the ALRs with reference microbial gene 856 vs. the exact logratio distances, corresponding to the Procrustes correlation of 0.9991. The number of distances plotted = 89 × 88/2 = 3, 916.

To visualize how close the ALR variables are to being isometric, Figure 2 shows all between-sample distances computed on the ALRs plotted against the corresponding exact logratio distances based on either all pairwise logratios or, equivalently, on all CLRs.

**Figure 3:**
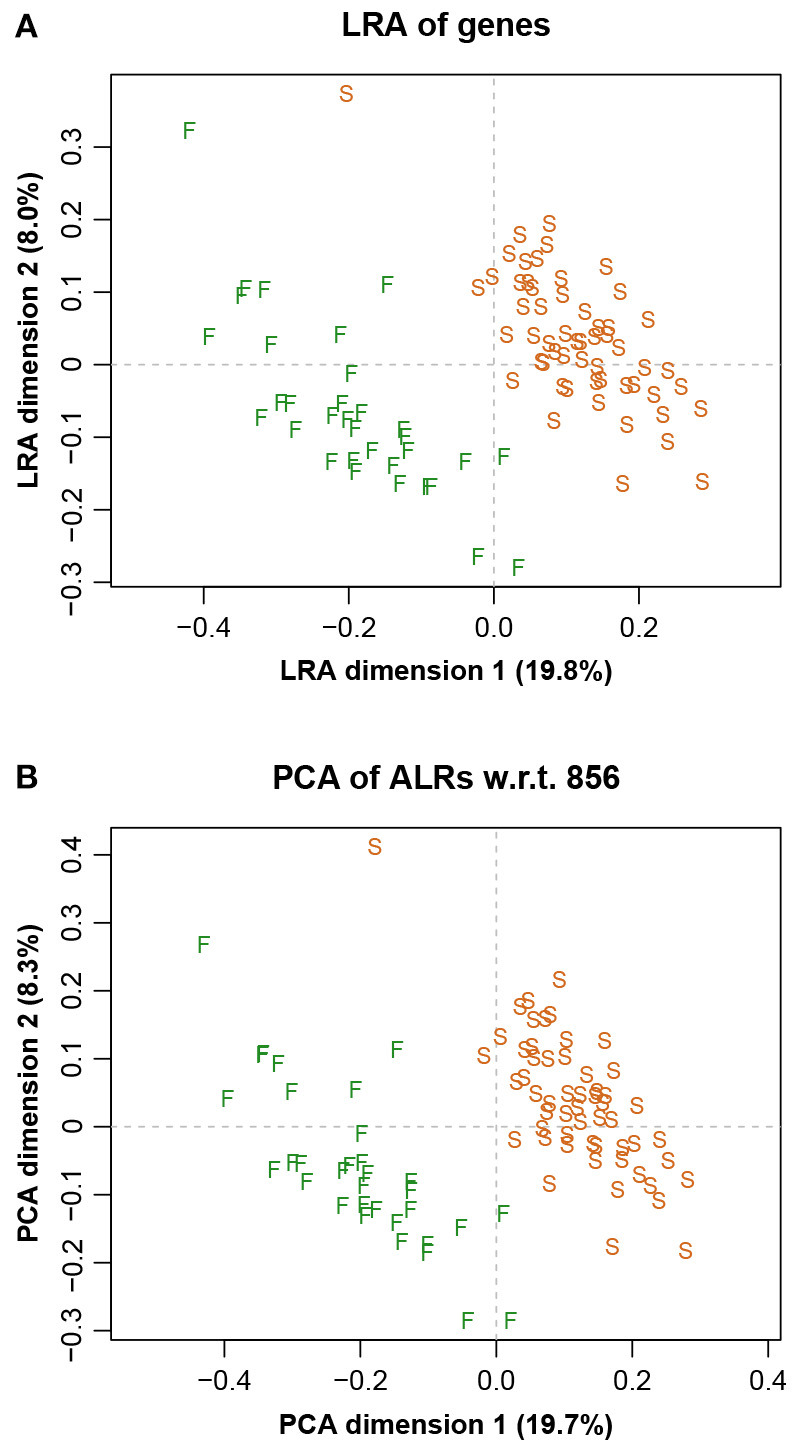
**(A)** Logratio analysis of the Rabbits data, aiming to explain the total logratio variance. **(B)** Principal component analysis of the additive logratios with reference component microbial gene number 856, showing a geometry practically identical to the exact logratio geometry (Procrustes correlation = 0.9991). The two groups of points are due to two sequencing laboratories, indicated here by F and S.

The LRA of the full dataset, showing just the samples, is shown in Figure 3A, while the corresponding PCA of the ALRs with reference gene 856 is shown in Figure 3B. They are practically identical, with very slight differences, as expected. The letters S and F stand for the two laboratories that did the sequencing, showing a clear separation. This sequencer effect was subsequently eliminated in the data analysis (Martínez-Álvaro et al., 2021b).

**Figure 4:**
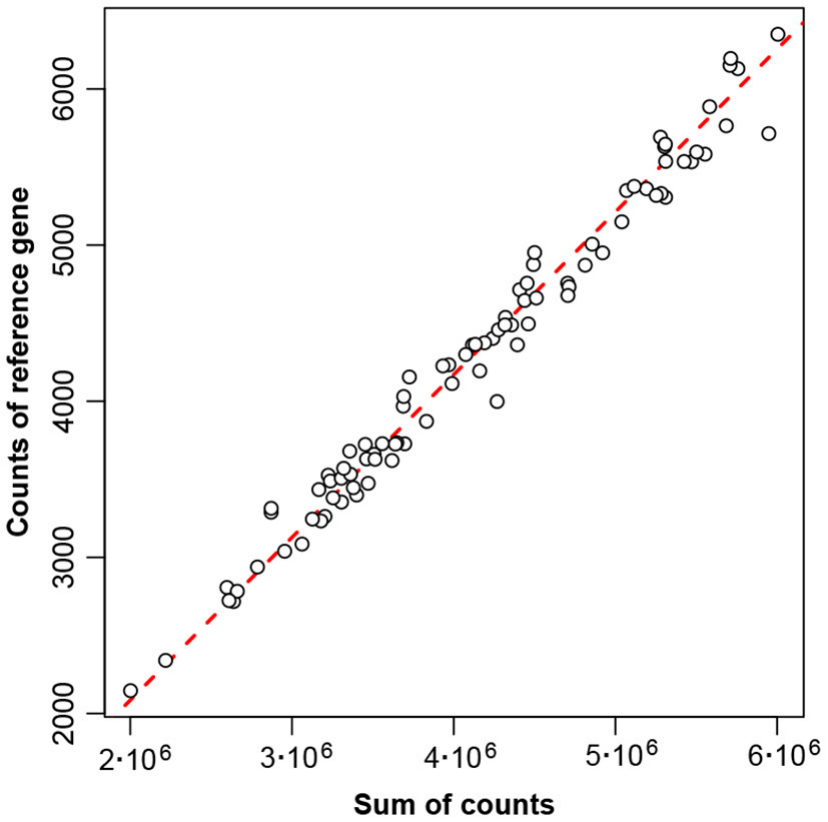
Proportionality between counts of reference gene number 856 and sum of counts, for the 89 samples of the Rabbits data. The diagonal line of exact proportionality goes through the origin (0,0).

The low variance of the reference gene means that in the original table of counts this gene's counts are closely proportional to the total counts—Figure 4 shows this conclusively.

It is interesting that the top candidates in this data set coming up as reference microbial genes are associated with the genetic machinery of the microbes, which are intrinsic in all microbial ecosystems. The same pattern has been found for other functional microbiome datasets (Martínez-Álvaro et al., 2021a).

### The Mice Data

This is a 28 × 3, 147 dataset of counts. There are 34 zeros in this dataset, which have been replaced using the function **cmultRepl** in R package **zCompositions** (Martín-Fernández et al., 2012).

(a) Total logratio variance = 0.2099, computed on the 3,147 CLRs of the components (transcripts). Equivalently, by transposing the dataset and then treating the samples as components, the same result is obtained on the smaller set of 28 CLRs of the samples.
(b) The highest Procrustes correlation is equal to 0.9977, corresponding to transcript number 1,318.
(c) The lowest variance of the log-transformed relative abundances of the candidates as reference components is equal to 0.00415, corresponding to transcript number 1,557. Its five-point summary on the log-scale is

$$\begin{array}{l} \text{ minimum}=-8.32\text{ first quartile}=-8.22\\ \text{ median}=-8.18\text{ third quartile}=-8.14\\ \text{maximum}=-8.03 \end{array}$$

showing again a high constancy in the values, with interquartile range of 0.08.

In this case the reference that maximizes the correlation is different from the one that minimizes the variance. One transcript, number 1,179, comes second on both criteria and is the one that was chosen, with Procrustes correlation = 0.9974 and variance = 0.00626. It has the 1617th highest relative abundance among the 3,147 transcripts, and its five-point summary is:


$$\begin{array}{l} \text{ minimum}=-9.69\text{ first quartile}=-9.62\\ \text{ median}=-9.57\text{ third quartile}=-9.50\\ \text{maximum}=-9.37 \end{array}$$

with interquartile range 0.12.

**Figure 5:**
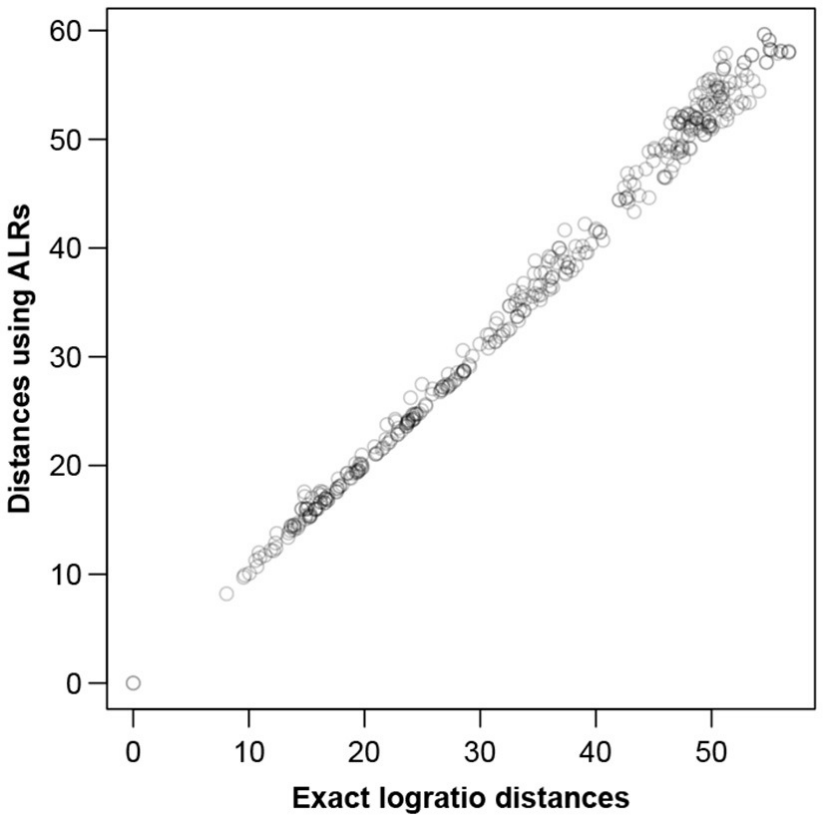
Between-sample distances for the Mice data based on the ALRs with reference transcript 1,179 vs. the exact logratio distances, corresponding to the Procrustes correlation of 0.9974. The number of pairs of distances plotted = 28 × 27/2 = 378.

To visualize how close the ALR transformation is to being isometric, Figure 5 shows the between-sample distances computed on the ALRs plotted against the exact logratio distances. The agreement is again excellent, with slightly less congruence in the high distances (commented below).

**Figure 6:**
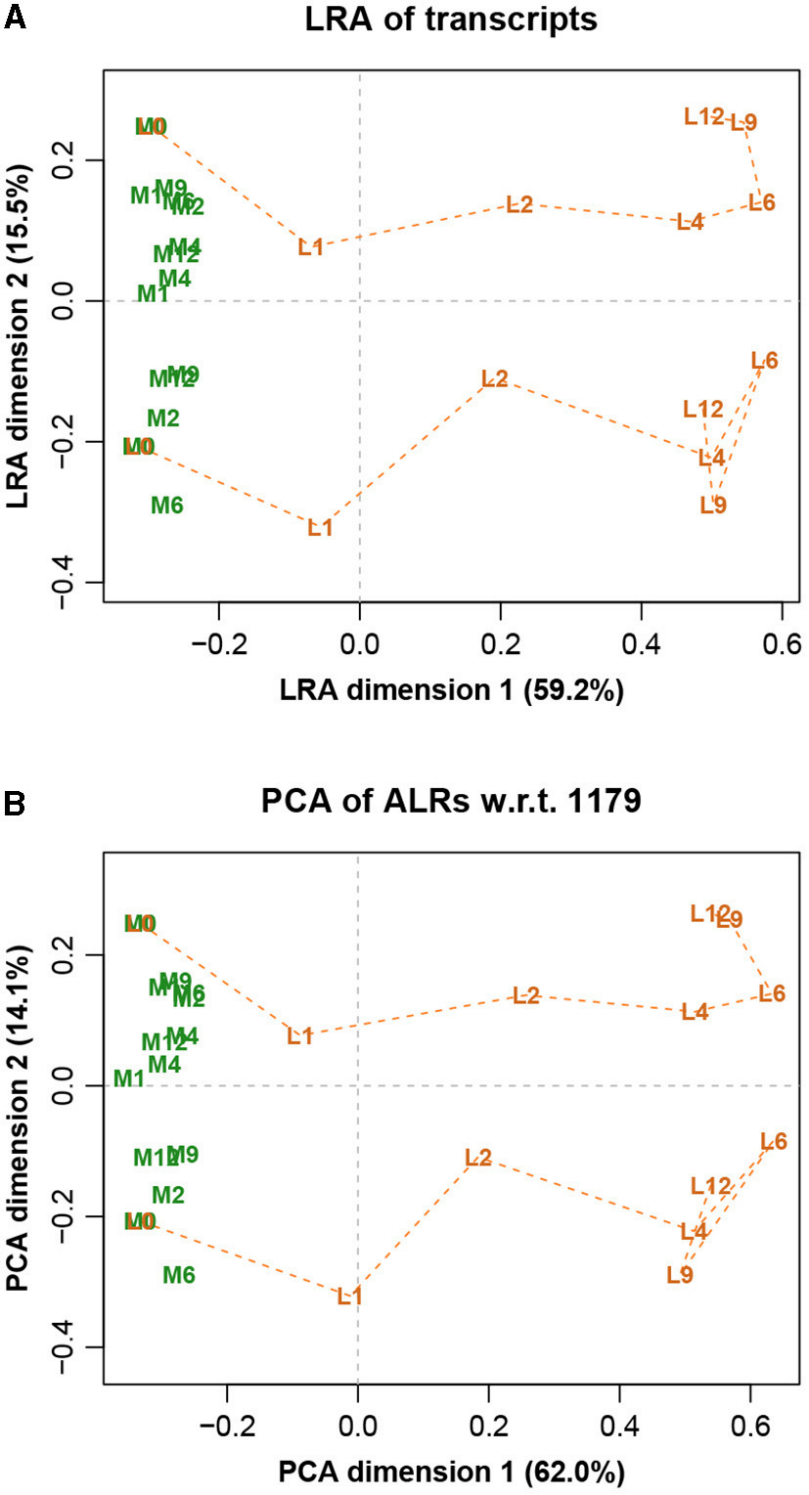
**(A)** Logratio analysis of the Mice data, aiming to explain the total logratio variance. **(B)** Principal component analysis of the additive logratios with reference transcript number 1,179, which has a geometry almost identical to the exact logratio geometry (Procrustes correlation = 0.9974). The label prefixes M and L refer to two treatments, and the number suffixes refer to times in hours. At time 0 the M and L trajectories are at the same points.

The LRA of the full dataset, showing just the samples, is shown in Figure 6A, while the PCA of the ALRs with reference transcript 1,179 is shown in Figure 6B. They are practically identical, with only very slight differences, again as expected from the very high Procrustes correlation. The labels stand for two different treatments (L and M) and 7 different times (0, 1, 2, 4, 6, 9, and 12 h). The slight discrepancies in the higher distances of Figure 5 correspond to the distances between samples of the different treatment groups, which are the most separated in Figure 6.

**Figure 7:**
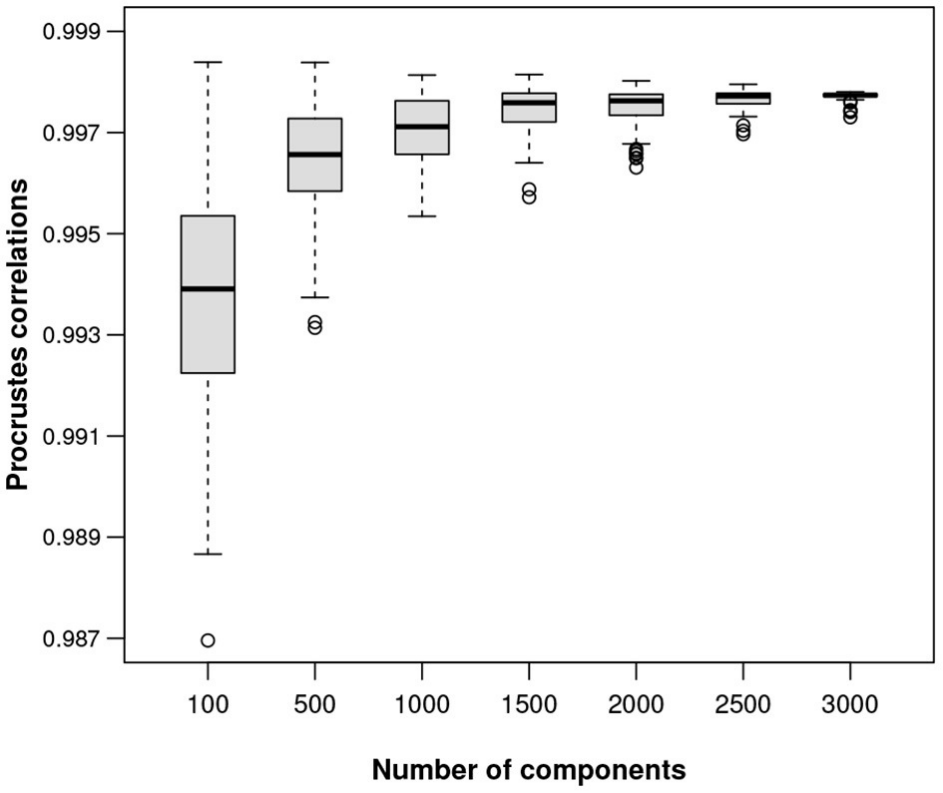
Boxplots of Procrustes correlations based on 100 random samples of the components (columns) of the Mice data set, for each of 100 up to 3,000 components.

In order to show the quality of the ALR transformations for data sets of any sizes, a simulation study was conducted on the Mice data, taking random samples of different sizes from the data, imagining each sample as a stand-alone one and finding the best reference for an ALR transformation for that particular data set. For subsets of 100, 500, 1,000, 1,500, 2,000, 2,500, 3,000, and 3,500 transcripts, and 100 random samples for each subset, the optimal Procrustes correlations are shown in the form of boxplots in Figure 7. As expected, the quality of the isometry of the ALR transformation improves as the number of possible reference components increases. In this particular example, even random samples of size 100 are doing well, with most references giving ALRs with Procrustes correlations over 0.99. The following example, with only 127 components in total, shows that the search for a near-isometric transformation using ALRs is still possible.

### The Cows Data

This is a 211 × 127 dataset of NMR intensities, measured as integrals (Bica et al., 2020). This dataset, which was provided with no zeros, originally had a few cases of zero integrals, which “were assumed to correspond to values below the limit of detection and were imputed based on the information from the other signals using the log-ratio expectation-maximization (EM) algorithm" (Štefelová et al., 2021). The samples were divided into three diet groups: concentrate, mixed or forage-based, and data on the methane yield was also measured.

(a) Total logratio variance = 0.09128 computed on the 127 CLRs of the components, which in this example are less than the number of samples. Notice that this value is lower than the first two data sets—this is not due to the fewer components, since our measurement of total logratio variance is an average, not a sum. It can be interpreted as the samples having less dispersion in this data set compared to the first two.
(b) The highest Procrustes correlation is equal to 0.9902, corresponding to the integral number 101 (labeled in the original dataset as Integral106). This component is the 26th highest in terms of relative abundance, out of the 127 integral components.
(c) The lowest variance of the log-transformed reference components is equal to 0.01115, corresponding to the integral number 109 (labeled in the original dataset as Integral115). Its five-point summary on the log-scale is:

$$\begin{array}{l} \text{ minimum}=-5.65\text{ first quartile}=-5.43\\ \text{ median}=-5.37\text{ third quartile}=-5.30\\ \text{maximum}=-4.94 \end{array}$$

with interquartile range 0.13, a value comparable to that for the Mice data.

However, the Procrustes correlation of integral number 109 was only 0.944, so it was decided to use integral number 101 as the reference part, which has a variance of its log-transformed relative abundances equal to 0.0563 and five-number summary on the log-scale of:


$$\begin{array}{l} \text{ minimum}=-6.00\text{ first quartile}=-5.52\\ \text{ median}=-5.36\text{ third quartile}=-5.18\\ \text{maximum}=-4.88 \end{array}$$

The interquartile range of 0.34 is now much higher than before, and so the ALRs should always be interpreted as pairwise logratios with respect to the reference, not as approximating the logarithms of the numerator components as in the first two examples. The Procrustes correlation almost equal to 1 again means that the ALRs are, for all practical purposes, isometric.

**Figure 8:**
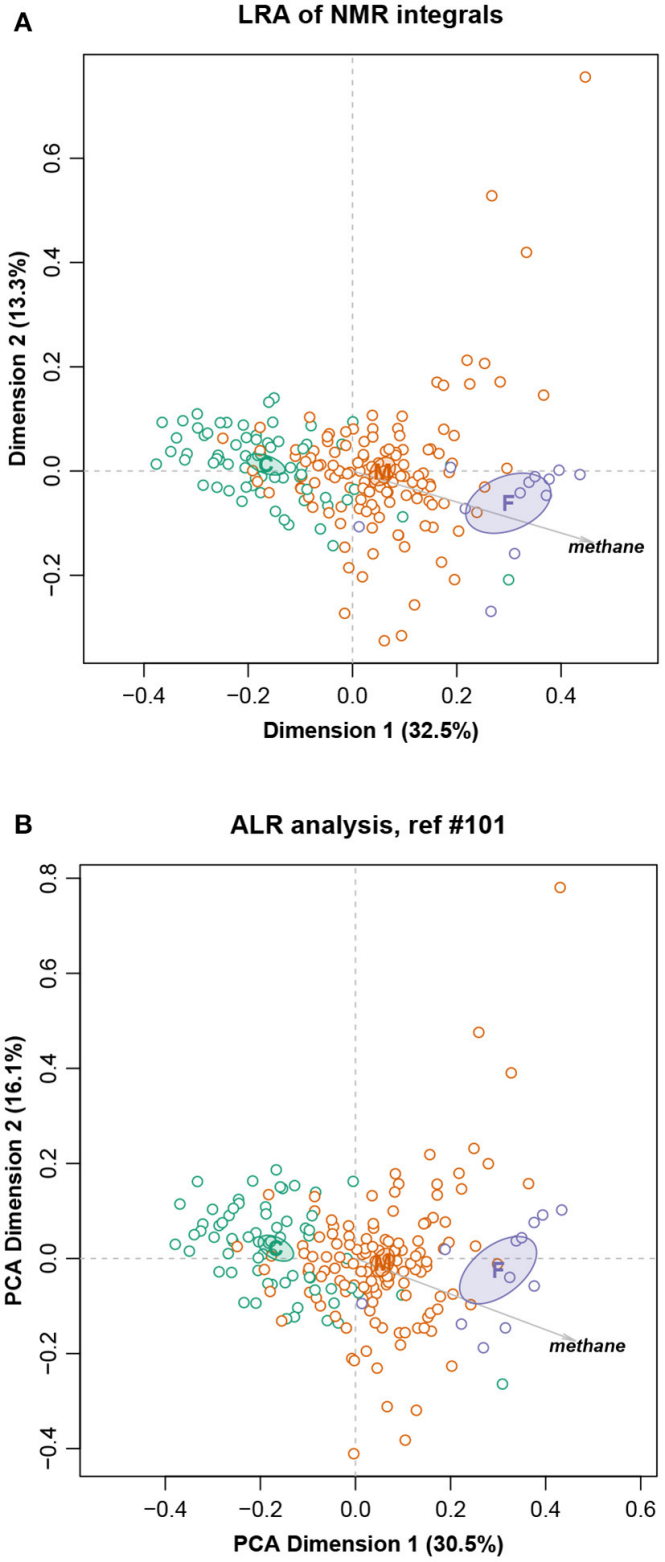
**(A)** Logratio analysis of the Cows data; **(B)** Principal component analysis of the additive logratios with reference integral number 101. Ninety-five percent confidence ellipses are shown for the three group means that are situated at the diet labels C (concentrate), M (mixed) and F (forage), as well as the supplementary variable methane. The geometry of the ALRs has a Procrustes correlation of 0.9902 with the exact geometry of all pairwise logratios.

To demonstrate again the almost exact isometry, Figure 8 shows the LRA using all the pairwise logratios (i.e., the PCA of the centered logratios), and the corresponding solution using the chosen set of ALRs. There are, once more, very small differences between the two solutions if one compares the configuration of the points in each case and the 95% confidence ellipses for the group means. The fact that these ellipses are well separated bears testimony to the highly significant differences between them (Greenacre, 2016). In addition, the directions of the supplementary methane variable in the two solutions are practically the same. As in the previous examples (Figures 3, 6), the percentages of variance displayed in the two-dimensional reduced spaces are similar: 45.8% for the LRA and 46.6% for the PCA of the ALRs.

## Discussion

Our objective has been to show that the ALR transformation, the simplest one in the CoDA toolbox, can provide a valid solution for the analysis of high-dimensional compositional datasets. The challenge is to find a good reference part.

In the Rabbits and Mice datasets with more than 3,000 components, there was more chance to find a reference component with the two desirable properties for constructing a set of ALRs. First and foremost, the reference has to result in a high Procrustes correlation between the exact logratio geometry and the ALR geometry, both of which have the same dimensionality. In all three examples, even the third one with much fewer components, we have found that the ALR transformation is suitable for representing the logratio variability, and has provided almost exact isometry with the logratio geometry. A secondary criterion, which is not a prerequisite but rather a fringe benefit, is low variance in the log of the relative abundance, which considerably simplifies the interpretation of the ALRs—this was satisfied for the two datasets with thousands of components, but not with the smaller Cows dataset.

There has been a rejection in the compositional data analysis literature of variables that are not exactly isometric in the mathematical sense, and variables that are “oblique”—see, for example, Hron et al. (2021). This criticism is difficult to understand when it is possible to come up with a set of variables that reproduces almost perfectly the logratio geometry, which means that the criticism is aimed at what is a near-zero lack of isometry. Notice that we are not claiming that this strategy will always work, but it has been successful in all the data sets that were easily available to us and which have been reported here, including 30 simulated datasets published in a recent article (see Supplementary Material). Since the benefit is great if this approach is indeed successful, it is recommended to try it as a first step in the compositional data analysis of such high-dimensional data. The method has been implemented by Martínez-Álvaro et al. (2021b) in an analysis of the Rabbits data and the near-isometric ALRs have been used to explain body fat characteristics of the sampled individuals. Coenders and Pawlowsky-Glahn (2020) show how to interpret logratios when used as explanatory variables in a linear regression model.

With respect to the ALRs, which are of concern here, these are simple pairwise logratios with respect to a chosen reference. If one is fortunate to find a reference that is almost constant in its relative abundance, this means that the pairwise logratio in each ALR is, for all practical purposes of interpretation, the same as the logarithm of the numerator. This makes the interpretation of the ALRs much easier when it comes to judging which ALRs are important for explaining variance, relating to covariates or distinguishing between groups.

We have shown that the ALR transformation can validly be used for high-dimensional datasets, and considerably simplifies the life of practitioners. The ALRs have a clear meaning, as opposed to the various complex logratio transformations that have generally been promoted, involving ratios of geometric means of components. The contrast between the simplicity of the ALR transformation, giving almost exact isometry, and other more complicated and less interpretable transformations, aimed at satisfying mathematically exact isometry, is evident—for example, in the recent re-analysis of the Cows data using a set of “weighted pivot logratios" (Štefelová et al., 2021), which is an isometric transformation involving geometric means along with a complicated weighting system. As far as weighting is concerned, this concept has existed for compositional data analysis since the mid-1970s in the work of Lewi (1976, 1986, 2005), who proposed default weights equal to the average relative abundances of the compositional components. Weighting the components is a trivial addition to compositional data analysis, as shown by definitions (Equations 2, 5), but can have substantial consequences when low abundance parts have high logratio variances due to measurement error (Greenacre and Lewi, 2009).

Hron et al. (2021) state that “alr coordinates cannot be simply identified with the individual original components, as they are in fact logratios, but the link with these is more clearly stated.” We have shown that this sweeping statement is in fact not true in some cases. When the reference is almost constant, then the components in the numerators of the ALRs are very close to being directly interpretable as the log-transformed relative abundances of the respective components. Then, for all practical purposes, the ALRs can be referred to as the components themselves. In addition, variances and correlations of the ALRs can be identified approximately with those of the numerators, apart from an overall scale factor, which makes the interpretation much easier. This simplification in the interpretation has been possible for our first two datasets, which have thousands of components, with the caveat that for the third set of NMR integrals, which has fewer components, the reference part does not have sufficiently low variance for this simplified interpretation, and thus the ALRs should be interpreted in that case as true ratios.

## Potential Implications

Our approach can make compositional data analysis simpler for the practitioner dealing with high-dimensional data, whereas much of the development in this area is, in our opinion, complicating its practice. The issues of spurious correlations, subcompositional incoherence and lack of isometry surely exist, but are usually raised in the context of modest data sets with few components. In the omics area these issues become diluted in compositions based on hundreds or thousands of components. We have hoped to show that for such high-dimensional data, the practitioner who wishes to follow the logratio approach can probably fall back on the simplest of logratio transformations, the additive logratios, with the benefit of their easy interpretation. This depends, of course, on finding a suitable reference component, which needs to be investigated for each new application. Following the strategy that we have laid out, the chances of finding a suitable additive logratio transformation appear to be high when there are very many potential reference components to choose from.

## Data Availability Statement

The Rabbits dataset will be available soon at https://www.ebi.ac.uk/ena/browser/view/PRJEB46755, with accession number PRJEB46755.

The Mice dataset is available at the repository http://doi.org/10.5281/zenodo.3270954 see Quinn (2018).

The Cows dataset was provided on request from the co-authors of (Bica et al., 2020) — see Acknowledgments.

The 30 sets of simulated data, analysed in the Supplementary Material, can be obtained from the supplementary material of Lloréns-Rico et al. (2021).

Other datasets and scripts can be downloaded from the github site of (Greenacre, 2018): https://github.com/michaelgreenacre/CODAinPractice, including the following:

- An R function **FINDALR** in the file **FINDALR.R** for computing the Procrustes correlation for all sets of ALRs using every possible component as reference, and identifying the largest one. This function will eventually be incorporated in the easyCODA package.
- An R script **Frontiers**_**ALR.R** for analysing the Mice dataset, including replacing the few data zeros. The other data sets are analysed in exactly the same way, even more simply since they have no data zeros.
- An R script **Frontiers** _**ALR** _**supplementary** for analysing the two supplementary applications to real data, using both the unweighted and weighted logratio options, as well as focusing on the subspace of the sample groups in the case of the first example.
- The two data sets for the Supplementary Material.

The programming language was R (R Core Team, 2021), with packages **easyCODA** (Greenacre, 2018), **vegan** (Oksanen et al., 2019), installed automatically with **easyCODA)** and **zCompositions** (Martín-Fernández et al., 2012).

Using a Toshiba Satellite S70 laptop, the time taken to compute the optimal reference part was 2090 secs (34.8 minutes) for the Rabbits data (3937 components), 77 secs for the Mice data (3137 components, but a lower sample size, which impacts significantly on the time) and 7 secs for the Cows data (127 components). Timings for the Supplementary Material examples are reported in the corresponding script.

## Author Contributions

MG conceived the manuscript based on an idea by MM-Á, and wrote the first draft. MM-Á and AB commented on the manuscript, made changes and provided additional references. MG added another example as supplementary material to reinforce the argument, did all the R coding. All authors discussed and revised the article several times and then approved the final version of the manuscript.

## Funding

Support is acknowledged from the Spanish National Plan of Scientific Research, Project PID2020-115558GB-C21.

## Conflict of Interest

The authors declare that the research was conducted in the absence of any commercial or financial relationships that could be construed as a potential conflict of interest.

## Publisher's Note

All claims expressed in this article are solely those of the authors and do not necessarily represent those of their affiliated organizations, or those of the publisher, the editors and the reviewers. Any product that may be evaluated in this article, or claim that may be made by its manufacturer, is not guaranteed or endorsed by the publisher.
