# Supplementary material for: Compositional Data Analysis of Microbiome and Any-Omics Datasets: A Validation of the Additive Logratio Transformation
Source: Front Microbiol. 2021 Oct 11;12:727398. doi: 10.3389/fmicb.2021.727398 (PMC8561721; doi:10.3389/fmicb.2021.727398)
Supplement: Supplementary file 1 [file Presentation_1.pdf]

Additional examples

Two additional applications, both to human data, are included here to support our argument that it may well be possible to find an additive logratio (ALR) transformation that approximates almost perfectly the logratio structure of a compositional data set, thereby considerably simplying the subsequent analysis and interpretation. The analytical context can be either unsupervised, looking for simple structure in the dataset, or supervised where compositional variables are sought to explain a response variable, which could be continuous or discrete. The code for these additional analyses are provided in an R script `Frontiers_ALR_supplementary.R`.

Finally, using 30 simulated data sets published by Lloréns-Rico et al. (2021), the selected ALR transformation is shown to behave well in every case, achieving Procrustes correlations higher than 0.99 in almost all cases.

1 HUMAN STOOL MICROBIOME

This is a dataset from the stool samples of $I = 490$ patients in a study of colono-rectal cancer, on which $J = 335$ microbiota operational taxonomic units (OTUs) were identified, referred to as the Cancer data (Baxter et al., 2016); the individuals in this dataset were classified as normal, adenoma (benign tumor) or cancer and the objective was to identify OTUs that discriminated between these groups, especially to predict the cancer group. This is a 490×335 dataset of counts. This dataset has a large number of zero counts (58% of the whole dataset) — a one was added to all the counts in the dataset before closing and taking logarithms. One OTU was removed due to a very large outlying value, which might be incorrect, reducing the dataset to 334 OTUs. The samples were divided into three groups: adenoma (benign tumor), cancer and normal.

This example illustrates several additional aspects:

- the use of a weighted analysis compared to the unweighted one, where the weighted analysis gives an improvement;
- the way to choose a reference component if interest is centered, as in this case, on the differences between the three sample groups.

In a first analysis, using the provided R function `FINDALR` the best Procrustes correlation obtained with the full-space geometry (of dimension 333, one less than the number of OTUs) was 0.936, using reference component . This result is lower than the three examples analyzed before. However, if the analysis is weighted, using the default weighting of (Lewi, 2005) and (Greenacre and Lewi, 2009), the solution is improved to a Procrustes correlation of 0.953, which is considered satisfactory for reproducing the full-space geometry.

However, since the objective of the data is to find the OTUs that discriminate between the groups, agreement in the geometry is not required in the full space but rather in the reduced space of the group means, excluding the dimensions not related to group differences. So, in the search for maximum agreement, a constrained (or restricted) LRA is performed on the complete set of CLRs, constrained to the three group means, and compared with the series of similarly constrained PCAs of ALR-transformed data using the respective reference components. Constrained PCA is called redundancy analysis (RDA) and available in the vegan package (Oksanen et al., 2019). In both cases we are not interested in variance that is not related to the distinction between the three sample groups. The results of this restricted search are as follows.

(a) Total logratio variance = 1.5301 computed on the 334 CLRs of the components, which in this example are less than the number of samples. Notice that this value is much higher than those of the three examples in the main text, which is typical of taxa datasets, with many zeros and larger contrasts in the data. However, the restricted between-group variance that we focus on here is small, equal to 0.0125, only 0.82% of the total. This restricted variance is lower than, but more in line with, the variances of the three previous examples. Nevertheless, the group differences are highly significant ($p < 0.0001$), using the multivariate permutation test also available in the R package vegan by Oksanen et al. (2019).

(b) The highest Procrustes correlation is equal to 0.9997, corresponding to the OTU number 312 (labelled in the original dataset as Otu000363). Its five-number summary is as follows:

minimum = -9.24 first quartile = -9.23 median = -9.18 third quartile = -8.13 maximum = -6.67

(c) The lowest variance of the log-transformed reference components is equal to 0.308, corresponding to OTU number 320 (labelled in the original dataset as Otu000372). Its five-point summary on the log-scale is:

minimum = -9.24 first quartile = -9.23 median = -9.22 third quartile = -9.18 maximum = -3.20

Since this OTU has only 36 nonzero values out of 490, the above estimated quartiles relate mostly to the overwhelming number of zeros, hence the small differences in quartiles up to the third, with positive skewness up to the maximum corresponding to the nonzero values.

It was decided to use this OTU number 312 as the reference part, with variance of its log-transformed relative abundances equal to 0.476 and many more nonzero abundances, 213 out of 490. Because of the variability in the reference part, the ALRs should always be interpreted as pairwise logratios with respect to the reference, not as approximating the logarithms of the numerator components.

The Procrustes correlation almost equal to 1 again means that the ALRs are, for all practical purposes, isometric, in this case isometric for group discrimination. Figure S1 shows the constrained solution of the between-group variance using all the pairwise logratios (i.e. in practice, the CLRs), and the corresponding solution using the ALRs. Because of the Procrustes correlation close to 1, there is no noticeable difference between the two solutions. The fact that the confidence ellipses for the groups means are highly separated bears testimony to the highly significant differences between them. For each axis two percentages are given: the first is the explained variance relative to the two-dimensional constrained logratio variance ($= 0.0125$), the second is the same explained variance relative to the much larger total logratio variance ($= 1.5301$).

In this example there were a large number of data zeros, typical of a microbial taxa dataset. These data zeros force samples onto the sides of the simplex space of the original compositions, and in the logratio transformations these become outliers, hence the difficulty in matching the exact and approximate geometries in the full space of the dataset. When constraining the solution to discriminate between the three groups, however, the outliers are much less important and so the ALRs, with the reference that was chosen to produce a configuration close to the constrained solution, has functioned surprisingly well, as shown in Figure S1.

Having made a selection of a set of ALRs that reproduces the between-group geometry, these ALRs can be used in a model for predicting the groups. Baxter et al. (2016) implemented a random forest prediction algorithm, combined with a backward elimination of the components, using the R package AUCRF (Calle et al., 2011) to get an optimal subset of OTUs. The classification is aimed at predicting the cancer and normal groups (hence the adenoma group is omitted). Using the relative abundances as inputs to the algorithm, this results in 33 OTUs being chosen, and an overall error rate of 22%, with 52% of the cancers

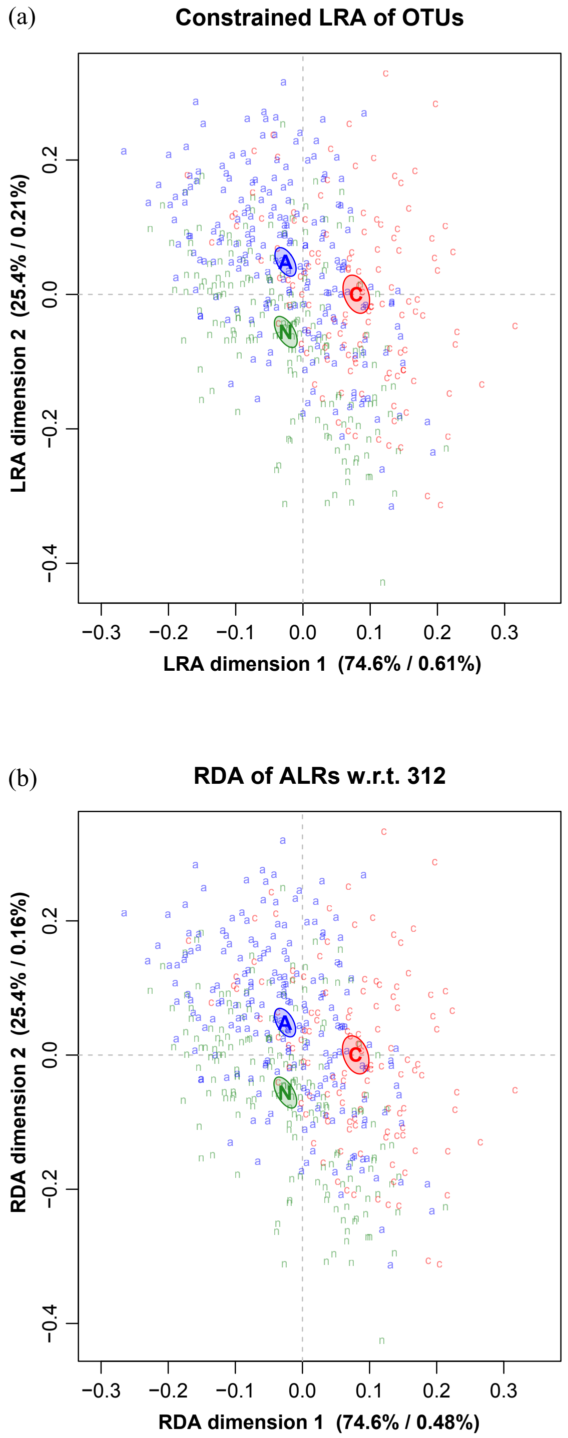

Figure S1. (a) Constrained logratio analysis of the Baxter data, aiming to explain the between-groups logratio variance. (b) Constrained principal component analysis (i.e. RDA) of the additive logratios with reference OTU number 312. In each case the first percentage expresses explained variance relative to the constrained logratio variance, whereas the second percentage is relative to the total logratio variance.

correctly predicted, and 97% of the normals. As a comparison, using the ALRs with respect to reference 856, the same algorithm with the same decision rule results in 34 OTUs being chosen, and an overall error rate of 20%, with 66% of the cancers correctly predicted and 90% of the normals. Moreover, the list of

chosen OTUs is very similar (i.e. in the case of the ALRs, the numerator OTUs), with the top 10 in each selection identical. At least in this example, using ALRs has performed slightly better, with the added value that logratios are being used, conforming to good practice in compositional data analysis.

The use of the relative abundances by Baxter et al. (2016) seems to be hardly different than the logratio approach, at least for the variable selection and prediction. Correspondence analysis (CA), which also operates on the relative abundances, has also been applied to this dataset (Greenacre, 2021) and shown to produce similar results. There is a theoretical reason underpinning CA, however, and that is the fact that the chi-square distance in CA has a close connection to logratio distance, and the issue of lack of subcompositional coherence is less critical (Greenacre, 2010, 2011b,a). The striking advantage of CA for compositional data analysis is the fact that it handles zero values naturally, which is in fact why it is so popular in ecology and other fields where data can be very sparse, such as archaeology and linguistics.

Finally, the whole exercise for reproducing the reduced-space geometry is repeated for weighted OTUs in the supplementary R script. The best Procrustes correlation is 0.9983, almost as good as the unweighted version, and again with excellent reproduction of the geometry. Whether to use the similarly performing unweighted or weighted versions would thus be based on considering the role of the OTUs in the solution, their measurement errors, the OTUs that are mainly contributing to the solution and their substantive interpretation.

2 HUMAN VAGINAL MICROBIOME

This dataset by (Deng et al., 2018) is cited and analysed by (Wu et al., 2021), an article alerted to us by one of the referees. The code for this analysis is again included in the supplementary R script. Applying our ALR selection, unweighted and weighted, was successful in both cases, and here the weighted version slightly improved the solution. In the unweighted case the best ALR transformation gave a Procrustes correlation of 0.969, whereas with weighting the Procrustes correlation increased to 0.983. The comparison of the two-dimensional geometries of the exact case (all logratios) and approximate case (the ALRs) for the weighted analysis is shown in Figs. S2a and S2b. The only detectable difference is that the ALR map appears to be a bit “stretched” horizontally compared to the LRA map, as can also be assessed by comparing the percentages of variance on the principal axes. The total logratio variances in the two cases were (a) 3.258 and (b) 7.710, much higher than all the previous examples — this can also be seen by the ranges of the scales on the axes in Fig. S2 compared to those of the previous examples.

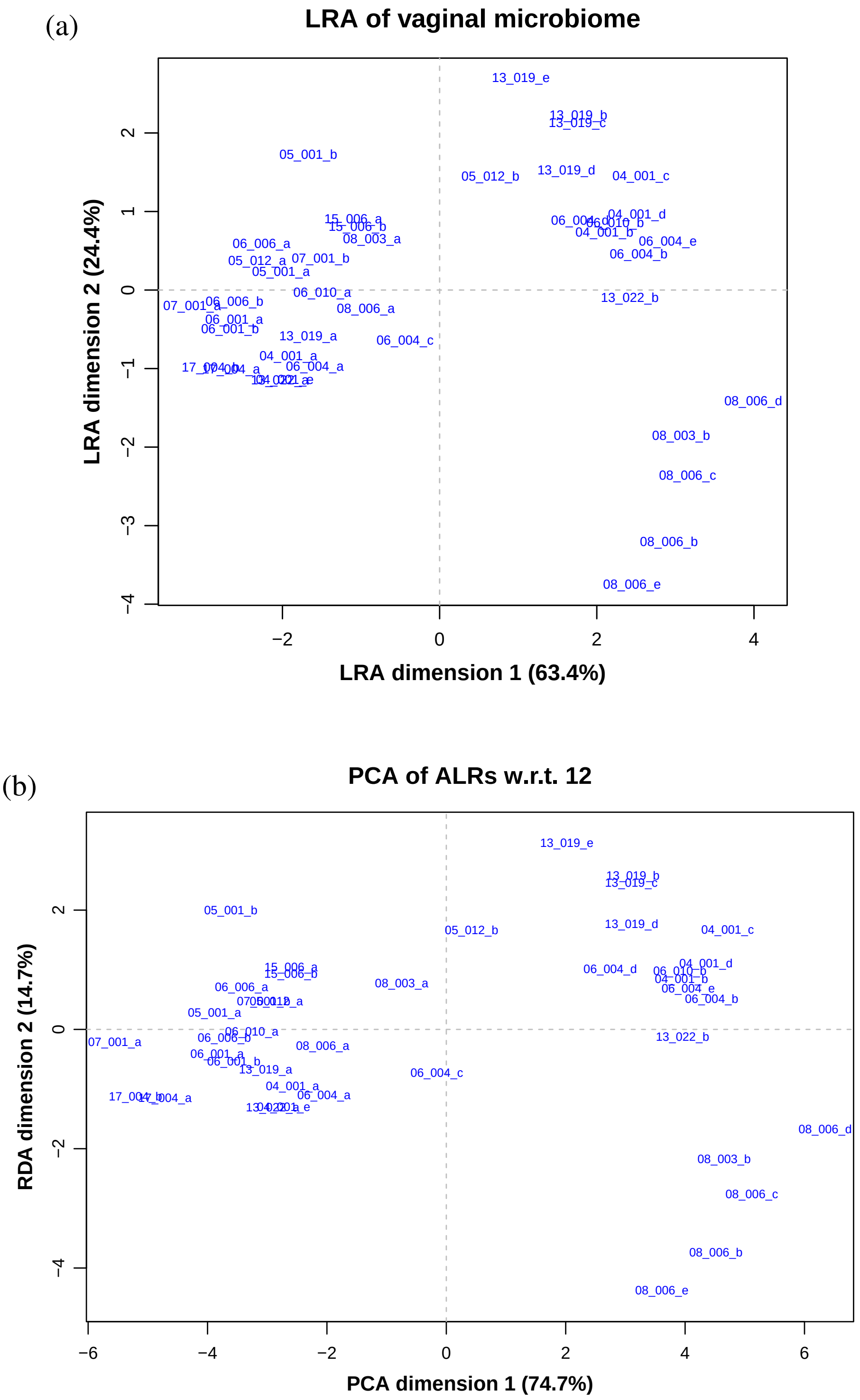

Figure S2. (a) Logratio analysis of the vaginal microbiome data. (b) Principal component analysis of the additive logratios with component reference number 12, which gives a Procrustes correlation with the exact logratio geometry of 0.983.

3 APPLICATIONS TO SIMULATED DATA

Lloréns-Rico et al. (2021) generated 30 simulated data sets, which are published in their supplementary material as an R workspace. These are 10 replicates of simulated data under each of three scenarios, Blooming, Dysbiosis and Succession, referred to here as B, D and S respectively (see the cited article for details). Each simulation was of counts of 300 taxa in 1000 samples, from which the authors took random samples of 200 to be more realistic of microbiome studies. We did the same, random sampling 200 individuals from each of the 30 data sets, and in each case finding the optimal ALR transformation that maximized the Procrustes correlation with the exact logratio geometry. The results are shown in Fig. S3, demonstrating the success of the approach in every single case. All correlations, except three in scenario D, are above 0.99 and a few are even over 0.995.

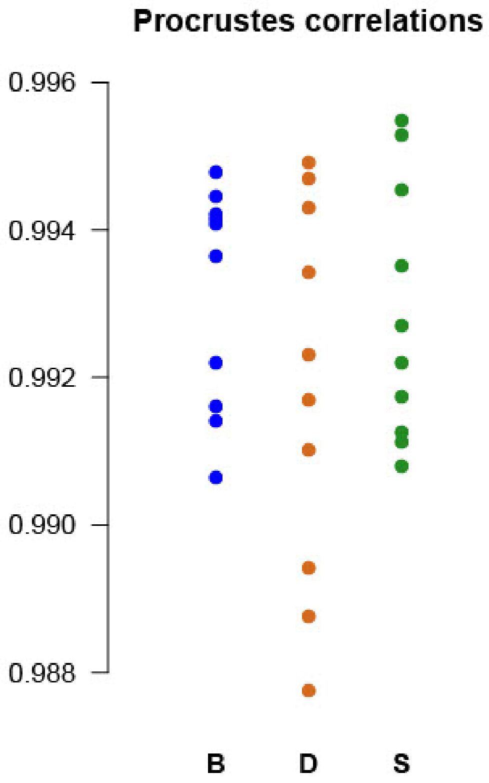

Figure S3. The results of applying the optimal ALR transformation to 30 different data sets, 10 simulated under each of three different scenarios, B, D and S. Dot plots are shown of the 30 maximized Procrustes correlations.

REFERENCES

Baxter, N., Ruffin, M., Rogers, M., and Schloss, P. (2016). Microbiota-based model improves the sensitivity of fecal immunochemical test for detecting colonic lesions. *Genome Med* 8. doi:10.1186/s13073-016-0290-3

Calle, M., Urrea, V., Boulesteix, A.-L., and Malats, N. (2011). Auc-rf: A new strategy for genomic profiling with random forest. *Hum Hered* 72. doi:121--32

Deng, Z.-L., Gottschick, C., Bhuju, S., Masur, C., Abels, C., and Wagler-Döbler, I. (2018). Metatranscriptome analysis of the vaginal microbiota reveals potential mechanisms for protection against metronidazole in bacterial vaginosis. *mSphere* 3

Greenacre, M. (2010). Log-ratio analysis is a limiting case of correspondence analysis. *Math Geosc* 42, 129–34

Greenacre, M. (2011a). Compositional data and correspondence analysis. In *Compositional Data Analysis: Theory and Applications*, eds. V. Pawlowsky-Glahn and A. Buccianti (New York: Wiley). 104–13

Greenacre, M. (2011b). Measuring subcompositional incoherence. *Math Geosc* 43, 681–93

Greenacre, M. (2021). Compositional data analysis. *Annu Rev Stat Appl* 8, 271–99

Greenacre, M. and Lewi, P. (2009). Distributional equivalence and subcompositional coherence in the analysis of compositional data, contingency tables and ratio-scale measurements. *J Classif* 26, 29–54

Lewi, P. (2005). Spectral mapping, a personal and historical account of an adventure in multivariate data analysis. *Chem Intell Lab Syst* 77, 215–23

Lloréns-Rico, V., Vieira-Silva, S., Gonçalves, P., and et al. (2021). Benchmarking microbiome transformations favors experimental quantitative approaches to address compositionality and sampling depth biases. *Nat Commun* 12, 3562

Oksanen, J., Blanchet, F., Friendly, M., Kindt, R., Legendre, P., McGlinn, D., et al. (2019). *vegan: Community Ecology Package. R package version 2.5-6*

Wu, J., Macklaim, J., Genge, B., and Gloor, G. (2021). Finding the centre: compositional asymmetry in high-throughput sequencing datasets. In *Advances in Compositional Data Analysis*, eds. P. Filzmoser, K. Hron, J. Martín-Fernández, and J. Palarea-Albaladejo (New York: Springer). 329–46
